# Antioxidant and Anti-Inflammatory Effects of Bioactive Compounds in Atherosclerosis

**DOI:** 10.3390/ijms26031379

**Published:** 2025-02-06

**Authors:** Ştefan Horia Roşian, Ioana Boarescu, Paul-Mihai Boarescu

**Affiliations:** 1 “Niculae Stăncioiu” Heart Institute Cluj-Napoca, 400001 Cluj-Napoca, Romania; dr.rosianu@gmail.com; 2 Cardiology Department of Heart Institute, “Iuliu Haţieganu” University of Medicine and Pharmacy Cluj-Napoca, 400001 Cluj-Napoca, Romania; 3 Neurology Department of Clinical Emergency County Hospital Saint John the New in Suceava, 720229 Suceava, Romania; 4 Department of Medical-Surgical and Complementary Sciences, Faculty of Medicine and Biological Sciences, “Ștefan cel Mare” University of Suceava, 720229 Suceava, Romania; paul.boarescu@usm.ro; 5 Cardiology Department of Clinical Emergency County Hospital Saint John the New in Suceava, 720229 Suceava, Romania

**Keywords:** atherosclerosis, antioxidant, anti-inflammatory, bioactive compound

## Abstract

Atherosclerosis, a chronic inflammatory disease characterized by the accumulation of lipids and immune cells within arterial walls, remains a leading cause of cardiovascular morbidity and mortality worldwide. Oxidative stress and inflammation are central to its pathogenesis, driving endothelial dysfunction, foam cell formation, and plaque instability. Emerging evidence highlights the potential of bioactive compounds with antioxidant and anti-inflammatory properties to mitigate these processes and promote vascular health. This review explores the mechanisms through which bioactive compounds—such as polyphenols, carotenoids, flavonoids, omega-3 fatty acids, coenzyme Q10, and other natural compounds—modulate oxidative stress and inflammation in atherosclerosis. It examines their effects on key molecular pathways, including the inhibition of reactive oxygen species (ROS) production, suppression of nuclear factor-κB (NF-κB), and modulation of inflammatory cytokines. By integrating current knowledge, this review underscores the therapeutic potential of dietary and supplemental bioactive compounds as complementary strategies for managing atherosclerosis, paving the way for future research and clinical applications.

## 1. Atherosclerosis

Atherosclerosis remains a leading cause of morbidity and mortality worldwide, underlying major cardiovascular events such as myocardial infarction, stroke, and peripheral artery disease. Traditionally considered a lipid storage disorder, atherosclerosis is now recognized as a multifactorial disease involving dyslipidemia, chronic inflammation, endothelial dysfunction, and immune dysregulation [1].

Endothelial dysfunction is widely recognized as an early and critical event in the pathogenesis of atherosclerosis [2,3,4,5]. It is characterized by impaired endothelium-dependent vasodilation, increased oxidative stress, a pro-inflammatory state, and a procoagulant phenotype [3,4,6]. Several mechanisms link endothelial dysfunction to the development and progression of atherosclerosis. First of all, oxidative stress is a key driver of endothelial dysfunction, leading to reduced nitric oxide bioavailability and impaired vasodilation [7]. Mitochondrial dysfunction in endothelial cells also contributes to oxidative stress and endothelial dysfunction, thereby promoting atherosclerosis [8,9]. Additionally, endothelial cell apoptosis and damage are thought to be early events in the atherosclerotic process [10]. Endothelial progenitor cells (EPCs) play an important role in endothelial repair and maintenance, and their dysfunction or depletion is associated with atherosclerosis [11,12]. Circulating markers of endothelial dysfunction, such as endothelial microparticles and platelet microparticles, have also been linked to atherosclerosis [13]. Inflammatory mediators, such as tumor necrosis factor-alpha (TNF-α), can further exacerbate endothelial dysfunction and contribute to the development of atherosclerosis [4,14]. Dyslipidemia, particularly hypertriglyceridemia, has also been shown to impair endothelial function and promote atherosclerosis [15,16].

Chen et al. emphasize the role of exosomal long noncoding RNA GAS5 in regulating apoptosis in endothelial cells and macrophages, suggesting that these cellular responses are crucial in the progression of atherosclerosis [17]. Furthermore, hyperuricemia has been shown to induce endothelial dysfunction through the activation of the HMGB1/RAGE signaling pathway, linking metabolic disturbances to vascular health [18].

The available evidence strongly supports the central role of endothelial dysfunction in the initiation and progression of atherosclerosis. Targeting the underlying mechanisms of endothelial dysfunction, such as oxidative stress, mitochondrial dysfunction, and inflammation, may represent promising therapeutic strategies for the prevention and treatment of atherosclerosis [19,20].

The prevalence and progression of atherosclerosis are significantly influenced by various risk factors, including age, hypertension, diabetes mellitus, and dyslipidemia. Studies have demonstrated that the burden of cardiovascular risk factors correlates with the severity of atherosclerosis [21].

An in vivo study performed by Boarescu et al. concluded that hypertension and dyslipidemia can rapidly induce reversible pro-atherogenic changes, characterized by the accumulation of lipid droplets in the subendothelial connective tissue. In contrast, type 1 diabetes mellitus serves as a significant cardiovascular risk factor that predominantly disrupts oxidative stress parameters, increases aortic levels of pro-inflammatory cytokines, and drives irreversible pro-atherogenic changes within the aortic wall [22].

Ihle-Hansen et al. found that the prevalence of carotid plaques in older populations is associated with the management of these risk factors over time, indicating that effective risk factor control can mitigate the extent of atherosclerosis [23]. Additionally, Jayachandran and Qu highlighted that conditions such as diabetes and obesity are among the most studied risk factors, contributing to the pathogenesis of atherosclerosis through mechanisms such as lipid accumulation and inflammation [24].

The biochemical modifications of LDL particles also play a significant role in atherosclerosis. Alique et al. discuss how oxidative stress and aging lead to alterations in LDL, which can exacerbate endothelial damage and promote atherosclerotic changes [25]. This is supported by findings from Baligh et al., who note that the multiplicity of vascular risk factors can significantly impact the severity of carotid atherosclerosis, suggesting that a comprehensive approach to managing these factors is essential for preventing disease progression [26].

High-density lipoprotein (HDL) has been widely recognized for its atheroprotective properties. However, recent research has highlighted the complexity of HDL’s role in atherosclerosis, particularly focusing on oxidized HDL (Ox-HDL) and dysfunctional HDL [27].

HDL is traditionally viewed as a protective factor against atherosclerosis due to its ability to facilitate the efflux of cholesterol from macrophages and other cells, thereby preventing foam cell formation and plaque development [28]. However, the functionality of HDL can be compromised by various factors, including oxidative stress, leading to the formation of Ox-HDL. Oxidation alters the structure and function of HDL, diminishing its protective effects and potentially converting it into a pro-atherogenic particle [29]. For instance, Ru et al. demonstrated that Ox-HDL accelerates atherosclerosis progression by inducing an imbalance between effector and regulatory T cells and relative gene expressions in LDL receptor knockout mice, highlighting the immunological implications of Ox-HDL in atherosclerosis [30].

The concept of dysfunctional HDL encompasses a range of alterations that impair its protective functions. Functionally defective HDL can act as a pro-oxidant, contributing to the production of reactive oxygen species (ROS) in monocytes and macrophages, which are pivotal in atherogenesis [31]. This shift from atheroprotective to pro-atherogenic properties is further supported by findings from Speer et al., who reported that abnormal HDL induces endothelial dysfunction via activation of Toll-like receptor-2, a key player in inflammatory responses [32]. Such dysfunctional HDL not only loses its vasoprotective properties but also promotes inflammatory processes that exacerbate atherosclerosis.

The structural modifications of HDL, particularly the oxidative modifications of apolipoproteins, play a significant role in the pathogenesis of atherosclerosis. For example, Huang et al. identified that myeloperoxidase (MPO) binds to HDL and is mechanistically linked to oxidant stress and atherosclerosis [33]. This interaction is critical, as MPO is released from activated leukocytes during inflammation, linking HDL dysfunction to the inflammatory milieu characteristic of atherosclerosis. Furthermore, studies have shown that HDL from patients with cardiovascular disease exhibits a distinct proteomic signature, indicating that alterations in HDL composition are associated with increased cardiovascular risk [34].

The clinical implications of dysfunctional HDL are profound, as they suggest that traditional approaches to increase HDL cholesterol may not adequately address the underlying pathophysiology of atherosclerosis. Instead, a focus on restoring HDL functionality could provide a more effective therapeutic strategy. For instance, pharmacological interventions aimed at improving HDL function have shown promise in preclinical studies, indicating that enhancing the atheroprotective properties of HDL may mitigate cardiovascular risk more effectively than merely raising HDL cholesterol levels [35].

Smoking is linked to vascular endothelial dysfunction, primarily due to reduced nitric oxide bioavailability. This reduction is believed to result from smoking-induced oxidative stress and inflammation, which compromise endothelial function, leading to atherosclerosis [36].

Moreover, the relationship between chronic kidney disease (CKD) and atherosclerosis is increasingly recognized. CKD is an independent risk factor for cardiovascular diseases, including atherosclerosis, as it exacerbates traditional risk factors such as hypertension and dyslipidemia [37]. Studies indicate that patients with CKD exhibit a higher prevalence of atherosclerosis, underscoring the need for vigilant cardiovascular risk management in this population [37].

Stress, both psychological and physiological, plays a significant role in the development and progression of atherosclerosis. Chronic stress activates the hypothalamic–pituitary–adrenal axis and the sympathetic nervous system, leading to increased secretion of stress hormones such as cortisol, adrenaline, and noradrenaline. These hormonal changes can contribute to several pro-atherogenic mechanisms, including endothelial dysfunction, inflammatory activation, or lipid metabolism dysregulation [38].

Emerging evidence also points to the role of systemic inflammation and infections in atherosclerosis. For instance, the association between *Helicobacter pylori* infection and increased risk of carotid atherosclerosis has been documented, suggesting that chronic infections may contribute to vascular inflammation and plaque development [39]. Additionally, periodontitis has been identified as a potential risk factor for atherosclerosis, with inflammatory responses linked to both conditions [40].

### 1.1. Oxidative Stress and Atherosclerosis

Oxidative stress is characterized by excessive ROS production in the cells and tissues that the antioxidant system cannot neutralize. This imbalance can lead to damage to cellular components, including deoxyribonucleic acid (DNA), proteins, and lipids. Furthermore, excessive ROS production has been shown to trigger inflammatory processes, promoting the synthesis and release of pro-inflammatory cytokines [41].

Oxidative stress is increasingly recognized as a pivotal factor in the pathogenesis of atherosclerosis, as it disrupts the balance between pro-oxidants and antioxidants, leading to endothelial dysfunction [6,42]. The interplay between oxidative stress and atherosclerosis is complex, involving various mechanisms that contribute to endothelial dysfunction, inflammation, and plaque formation [43].

The generation of ROS has been implicated in the initiation and progression of atherosclerosis. Elevated levels of ROS can lead to the oxidation of low-density lipoprotein (LDL), resulting in the formation of Ox-LDL, which is atherogenic and promotes inflammatory responses within the arterial wall [44,45,46]. The oxidative modification of LDL is considered one of the earliest events in atherosclerosis, triggering a cascade of inflammatory processes that contribute to plaque development [46,47].

Research has shown that oxidative stress not only promotes the oxidation of lipoproteins but also enhances the inflammatory response in atherosclerosis. For instance, studies have demonstrated that the presence of Ox-LDL stimulates the expression of adhesion molecules on endothelial cells, facilitating the recruitment of monocytes and their subsequent transformation into macrophages, which are key players in plaque formation [48,49]. Additionally, the inflammatory milieu created by oxidative stress can lead to the apoptosis of vascular cells, further exacerbating the progression of atherosclerosis [42,50].

Therapeutic strategies aimed at reducing oxidative stress have shown promise in mitigating atherosclerosis. Antioxidants, such as vitamins C and E, have been investigated for their potential to ameliorate oxidative damage and improve vascular health [51,52]. Moreover, natural compounds like curcumin have been highlighted for their antioxidant properties, which may help reduce the risk of atherosclerosis by modulating oxidative stress and inflammation [48,49,53,54,55]. The use of herbal medicines and dietary interventions to enhance antioxidant defenses is an area of active research, with studies indicating that such approaches may effectively lower the incidence of atherosclerosis [56,57,58,59].

### 1.2. Inflammation and Atherosclerosis

The understanding of the mechanisms underlying inflammation in atherosclerosis has evolved significantly, revealing a complex interplay between various immune cells, cytokines, and lipid metabolism [60].

Inflammation in atherosclerosis is primarily driven by the accumulation of Ox-LDL within the arterial wall. Ox-LDL not only promotes the recruitment of inflammatory cells but also stimulates the release of pro-inflammatory cytokines, such as interleukin-6 (IL-6) and TNF-α, from various cell types, including macrophages and endothelial cells [61,62]. This inflammatory response is further exacerbated by the activation of Toll-like receptor 4 (TLR4), which has been shown to mediate the inflammatory responses induced by Ox-LDL [61,63]. The chronic presence of these inflammatory mediators contributes to endothelial dysfunction, a hallmark of early atherosclerosis, and promotes the formation of foam cells, which are lipid-laden macrophages that accumulate in atherosclerotic plaques [62,64].

The role of specific immune cells in atherosclerosis has also been extensively studied. Macrophages are critical players in the inflammatory process, as they not only engulf Ox-LDL but also secrete various cytokines that perpetuate inflammation and contribute to plaque instability [62,63]. Additionally, T helper 17 (Th17) cells have been implicated in atherosclerosis, as they produce pro-inflammatory cytokines that can exacerbate the inflammatory milieu within plaques [65]. The presence of these immune cells in atherosclerotic lesions is associated with increased plaque vulnerability and the risk of rupture, which can lead to acute cardiovascular events [66,67].

Moreover, the relationship between lipid metabolism and inflammation is crucial in the context of atherosclerosis. The accumulation of lipids, particularly cholesterol, in the arterial wall not only triggers inflammation but also alters the function of immune cells, leading to a vicious cycle of inflammation and lipid deposition [64,65]. The expression of the ATP-binding cassette transporter A1 (ABCA1) has been shown to link cholesterol accumulation with inflammatory responses, suggesting that targeting lipid metabolism may have therapeutic implications for managing atherosclerosis [64].

Recent therapeutic approaches have focused on targeting inflammation to mitigate atherosclerosis. For instance, the Canakinumab Anti-inflammatory Thrombosis Outcomes Study (CANTOS) demonstrated that targeting IL-1β with monoclonal antibodies significantly reduced cardiovascular events in patients with a history of myocardial infarction and elevated inflammatory markers [6,68]. This highlights the potential of anti-inflammatory therapies in addressing the underlying inflammatory processes in atherosclerosis.

**Figure 1:**
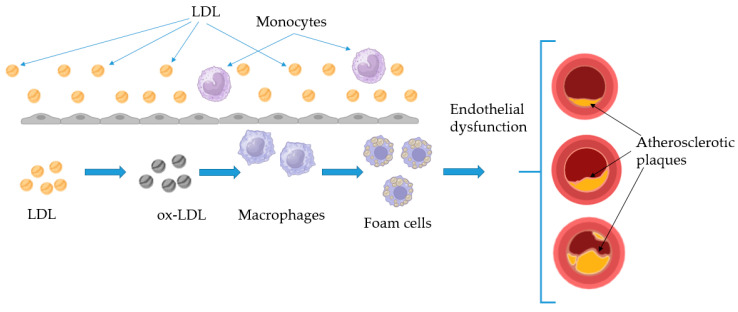
Pathogenesis of atherosclerosis.

To sum up, as shown in Figure 1, the pathogenesis of atherosclerosis begins with endothelial dysfunction, which allows LDL to accumulate and become ox-LDL in the intima, triggering an inflammatory response. Monocytes adhere to the endothelium, migrate into the intima, and differentiate into macrophages, which engulf oxidized LDL to form foam cells. These foam cells, along with T lymphocytes and cytokine release, amplify the inflammatory response, promoting smooth muscle cell migration, extracellular matrix deposition, and fibrous cap formation. Over time, plaques grow and may rupture, leading to thrombosis and complications, such as myocardial infarction or stroke.

## 2. Flavonoids

Flavonoids, a diverse group of polyphenolic have garnered significant attention for their potential antioxidant and anti-inflammatory effects in the context of atherosclerosis [69].

Flavonoids are abundant in various beverages and foods, including wine, beer, and tea, but are most concentrated in fruits, vegetables, flowers, and seeds. The levels of these compounds can vary depending on factors like plant variety or genotype, environmental growing conditions, soil characteristics, as well as harvest and storage practices [70].

Research has demonstrated that flavonoids can reduce oxidative stress by scavenging ROS and enhancing antioxidant defenses. For instance, flavonoids such as quercetin and catechin have been shown to inhibit LDL oxidation, a critical step in the initiation of atherosclerosis [71,72]. By preventing the oxidation of LDL, flavonoids help maintain endothelial function and reduce the inflammatory response associated with atherosclerosis. Additionally, studies indicate that flavonoids can modulate the expression of endothelial nitric oxide synthase (eNOS), leading to increased nitric oxide (NO) production, which is vital for vascular health [73], This modulation helps counteract the effects of oxidative stress and promotes vasodilation, thereby improving blood flow and reducing the risk of atherosclerosis. Epigallocatechin gallate (EGCG), found in green tea, exerts its protective effect by enhancing glucose uptake by promoting GLUT4 translocation to the skeletal muscle membrane. This mechanism involves the activation of the phosphoinositol 3-kinase (PI3K) and AMP-activated protein kinase (AMPK) signaling pathways, which are crucial in regulating glucose metabolism and energy balance in cells [74].

The anti-inflammatory properties of flavonoids are equally significant in the context of atherosclerosis. Flavonoids have been shown to inhibit the expression of pro-inflammatory cytokines, such as TNF-α and IL-6, which are crucial mediators in the inflammatory process of atherosclerosis [75,76]. For example, luteolin, a flavonoid derived from various plants, has been reported to attenuate the inflammatory response by modulating the signal transducer and activator of the transcription 3 (STAT3) signaling pathway, which is often activated in inflammatory conditions [76]. Furthermore, flavonoids can inhibit the activation of nuclear factor kappa B (NF-κB), a transcription factor that regulates the expression of numerous inflammatory genes [75,76]. This inhibition contributes to a reduction in the recruitment of inflammatory cells to the arterial wall, thereby mitigating plaque formation and progression [77].

In vivo studies have further substantiated the protective effects of flavonoids against atherosclerosis. For instance, a systematic review and meta-analysis indicated that flavonols significantly reduce aortic atherosclerosis lesion area in apolipoprotein E-deficient mice, highlighting their potential in modulating lipid profiles and inflammatory responses [78]. Additionally, higher habitual flavonoid intakes have been associated with a lower risk of peripheral artery disease, particularly in individuals with significant inflammation and oxidative stress [79]. These findings suggest that dietary flavonoids may play a crucial role in cardiovascular health by counteracting the detrimental effects of atherosclerosis [80].

Moreover, the mechanisms through which flavonoids exert their effects are multifaceted. They can influence various signaling pathways involved in inflammation and oxidative stress, including the modulation of cell adhesion molecules, such as vascular cell adhesion molecule 1 (VCAM-1) and P-selectin, which are critical for leukocyte adhesion and migration into the arterial wall By inhibiting these processes, flavonoids can reduce the inflammatory burden associated with atherosclerosis [71,81].

In clinical studies, dietary flavonoid intake was associated not only with beneficial changes in serum biomarkers related to HDL function in a variety of cardiovascular risk factors (e.g., hyperlipidemia, hypertension, or diabetes), including increased HDL cholesterol concentration but also with HDL antioxidant and cholesterol efflux capacities [82].

## 3. Polyphenols

Polyphenols are plant-derived micronutrients with essential physiological roles. They consist of diverse molecules containing one or more phenolic rings and are abundant in various foods, including wine, green tea, grapes, vegetables, red fruits, and coffee [83].

The role of polyphenols in mitigating atherosclerosis through their antioxidant and anti-inflammatory effects has gained considerable attention in recent years [84].

The consumption of polyphenol-rich foods has been associated with reduced inflammation and oxidative stress, both of which are critical factors in the pathogenesis of atherosclerosis. Polyphenols exhibit potent antioxidant properties, which are crucial in combating oxidative stress. They have been shown to enhance the body’s endogenous antioxidant defenses by upregulating antioxidant enzymes and reducing the production of ROS [85]. For instance, Andriantsitohaina et al. highlight that polyphenols regulate redox enzymes, thereby decreasing mitochondrial ROS production and improving vascular health [86]. Furthermore, Witkowska et al. emphasize that the beneficial effects of polyphenols on cardiovascular health may not solely stem from their antioxidant capacity but also from their ability to influence gut microbiota and enhance internal antioxidant mechanisms [87]. In addition to their antioxidant effects, polyphenols possess significant anti-inflammatory properties. Pounis et al. describe several mechanisms through which polyphenols can attenuate inflammation, including the inhibition of pro-inflammatory cytokines and the modulation of signaling pathways involved in inflammation [88]. This is corroborated by Wu et al., who note that polyphenols can reduce the secretion of pro-inflammatory markers, thereby mitigating low-grade inflammation associated with chronic diseases, including atherosclerosis [89]. The interplay between polyphenols and inflammation is further supported by Tangney and Rasmussen, who argue that the immunomodulatory effects of polyphenols may contribute significantly to their cardiovascular protective effects [90].

Epidemiological studies have consistently shown a correlation between polyphenol intake and reduced incidence of cardiovascular diseases. For example, Esposito et al. found that higher dietary polyphenol intake was associated with improved inflammatory markers and lipid profiles, which are very important in the context of atherosclerosis [91]. Similarly, Khurana et al. emphasize that the cardioprotective effects of polyphenols are evident in populations with diets rich in these compounds, suggesting a strong link between polyphenol consumption and cardiovascular health [92]. Moreover, Ziółkiewicz et al. specifically discuss the influence of polyphenols on atherosclerosis development, highlighting their multifaceted role in reducing oxidative stress and inflammation [93]. The mechanisms by which polyphenols exert their effects are complex and multifactorial. They not only act directly on endothelial cells to improve function and reduce inflammation but also influence gut microbiota, which in turn can modulate systemic inflammation and metabolic health [94]. This interaction underscores the importance of dietary patterns rich in polyphenols, such as those found in the Mediterranean diet, which have been linked to lower rates of atherosclerosis and related cardiovascular conditions [95].

A cross-sectional analysis performed by Castro-Barquero et al. concluded that there was an association between the intake of all polyphenol subclasses except phenolic acids and lignans, and higher HDL serum levels [96].

Curcumin, a polyphenolic compound derived from the turmeric plant, has been extensively studied for its therapeutic potential in various inflammatory [97,98,99] and cardiovascular diseases, including atherosclerosis [100,101,102,103]. The antioxidant and anti-inflammatory properties of curcumin are considered to be the primary mechanisms by which it exerts its protective effects against atherosclerosis [100,101,102,103].

Numerous in vitro and in vivo studies have demonstrated the ability of curcumin to suppress the inflammatory response in the context of atherosclerosis. Curcumin has been shown to inhibit the expression of pro-inflammatory mediators, such as C-reactive protein (CRP), TNF-α, IL-1β, and intercellular adhesion molecule-1 (ICAM-1), which play crucial roles in the development and progression of atherosclerosis [100,101,102,103,104,105].

The antioxidant properties of curcumin are also well-documented, as it can effectively scavenge various ROS and inhibit lipid peroxidation, which are key contributors to the pathogenesis of atherosclerosis [100,101,102,103,104,105]. Curcumin has been found to suppress the accumulation of Ox-LDL in macrophages, a critical step in the formation of foam cells and the development of atherosclerotic lesions [103,106,107].

Furthermore, curcumin has been shown to modulate lipid metabolism and improve the lipid profile, leading to a reduction in total cholesterol (TC), triglycerides, and LDL levels, while increasing high-density lipoprotein (HDL) levels. These effects on lipid metabolism contribute to the overall protective role of curcumin in atherosclerosis [103,108,109].

The anti-inflammatory and antioxidant properties of curcumin have also been linked to the activation of various signaling pathways, such as the nuclear factor-κB (NF-κB) and mitogen-activated protein kinase (MAPK) pathways, which are involved in the regulation of inflammatory and oxidative stress responses [100,101,103,110,111].

In addition to its direct effects on inflammatory and oxidative processes, curcumin has been reported to exert its atheroprotective effects through the modulation of other cellular and molecular mechanisms, such as the regulation of scavenger receptors and ATP-binding cassette transporters [106].

A recent randomized controlled trial that evaluated the effects of curcumin on reducing atherogenic risks in obese patients with type 2 diabetes concluded that it is promising for protecting against atherosclerosis and related cardiometabolic risks. Moreover, by reducing cardiometabolic risk factors, curcumin provides various health benefits, making it a valuable component of a balanced diet and healthy lifestyle [112].

## 4. Carotenoids

Carotenoids, a class of natural pigments found predominantly in plants such as pumpkins, carrots, parsnips, corn, or tomatoes, have garnered significant attention for their potential health benefits, particularly in the context of cardiovascular diseases and atherosclerosis. Carotenoids exhibit both antioxidant and anti-inflammatory properties, which may play a crucial role in mitigating the risk and progression of atherosclerosis [113].

Numerous studies have established a correlation between carotenoid intake and reduced cardiovascular risk. For instance, observational studies have consistently shown that higher plasma concentrations of carotenoids, such as lutein and lycopene, are associated with lower incidences of cardiovascular disease (CVD) and related mortality [113,114,115]. These findings are supported by systematic reviews that highlight the inverse relationship between dietary carotenoid levels and cardiovascular risk factors, including lipid profiles and inflammatory markers [114,116]. Specifically, carotenoids have been shown to scavenge reactive oxygen species ROS [117].

The antioxidant properties of carotenoids are attributed to their unique chemical structure, which allows them to interact with biological membranes and neutralize free radicals [117,118]. This action not only protects lipoproteins from oxidation but also helps maintain endothelial function [117,119]. For example, a study by Karppi et al. demonstrated that serum carotenoid levels were inversely related to the progression of early atherosclerosis in the carotid artery among men, suggesting a protective effect against vascular damage [120]. Furthermore, dietary intake of carotenoids has been linked to favorable lipid profiles, which are essential for cardiovascular health [114,116].

Additional properties of carotenoids that may contribute to reducing cardiovascular risk include their ability to lower blood pressure, decrease pro-inflammatory cytokines (IL-6) and inflammation markers (such as CRP), and enhance insulin sensitivity in muscle, liver, and adipose tissue [117]. The anti-inflammatory properties may further contribute to their cardiovascular benefits as inflammation is a significant factor in the development and progression of atherosclerosis, and carotenoids have been shown to modulate NF-κB pathway [121].

A post hoc analysis of the DIABIMCAP (Carotid Atherosclerosis in Newly Diagnosed Type 2 Diabetic Individuals) Study cohort concluded that a diet rich in fruits and vegetables, which results in higher plasmatic carotene concentrations, was associated with a lesser atherosclerotic plaque burden. In the same study, it was reported that no associations were found either between HDL and β- or total carotene plasma concentrations or between LDL or triglycerides and plasma carotenes [122].

Despite the promising evidence, the relationship between carotenoid supplementation and cardiovascular health remains complex. Some studies have reported inconsistent results, suggesting that the bioavailability of carotenoids and the presence of other dietary components may influence their effectiveness [114,123]. Moreover, intervention trials with isolated carotenoid supplements have often failed to replicate the protective effects observed with dietary intake, indicating that the matrix of whole foods may play a critical role in their health benefits [121,123].

## 5. Black Cumin (*Nigella sativa*)

*Nigella sativa*, also known as black cumin, has been extensively studied for its therapeutic potential in various inflammatory and cardiovascular diseases, including atherosclerosis [124]. The antioxidant and anti-inflammatory properties of *Nigella sativa* are considered to be the primary mechanisms by which it exerts its protective effects against atherosclerosis [125].

*Nigella sativa* has been shown to inhibit the expression of pro-inflammatory mediators, such as TNF-α, IL-1β, and monocyte chemoattractant protein-1 (MCP-1) [126,127,128].

The antioxidant properties of *Nigella sativa* are also well-documented, as it can effectively scavenge various ROS and inhibit lipid peroxidation [127,128,129,130]. *Nigella sativa* has been found to suppress the accumulation of LDL in macrophages [126].

Furthermore, *Nigella sativa* has been shown to modulate lipid metabolism and improve the lipid profile, leading to a reduction in TC, triglycerides, and LDL levels, while increasing HDL levels. It may act as a cholesterol-lowering agent by enhancing hepatic LDL receptor (LDLR) expression, which promotes the uptake of serum LDL-C, and by suppressing cholesterol synthesis through the inhibition of HMG-CoA reductase activity. These effects on lipid metabolism contribute to the overall protective role of *Nigella sativa* in atherosclerosis [131,132].

The anti-inflammatory and antioxidant properties of *Nigella sativa* have also been linked to the activation of various signaling pathways, such as NF-κB and MAPK pathways, which are involved in the regulation of inflammation and oxidative stress [133].

Long-term supplementation with *Nigella sativa* over one year significantly reduced TC, mean arterial pressure (MAP), and heart rate (HR) in a nonrandomized clinical trial with type 2 diabetic patients receiving oral hypoglycemic agents (OHA). These findings indicate that *Nigella sativa* seeds may serve as a valuable complementary therapy alongside other anti-atherogenic and antihypertensive medications for managing diabetic complications and might offer a preventive therapeutic strategy aimed at reducing CVD risk in this high-risk population [134].

A meta-analysis performed by Khodami et al. demonstrated the positive effect of *Nigella sativa* on LDL, HDL, and TC in most of the analyzed studies but did not show any signs of improvement among older patients [135].

## 6. Ginger (*Zingiber officinale*)

Ginger (*Zingiber officinale*), which is rich in phytocompounds 6-gingerol, 6-shogaol, and zingerone, besides other phenolics and flavonoids, has been studied for its therapeutic potential in various anti-inflammatory and antioxidant effects [136,137]. The antioxidant and anti-inflammatory properties of ginger are considered to be the primary mechanisms by which it exerts its protective effects against atherosclerosis [138].

In vitro and in vivo studies have demonstrated the ability of ginger to suppress the inflammatory response in the context of atherosclerosis. Ginger has been shown to inhibit the expression of pro-inflammatory mediators, such as TNF-α, IL-1β, and monocyte MCP-1, which play crucial roles in the development and progression of atherosclerosis [139,140,141,142].

Ginger enhances the activity of superoxide dismutase (SOD), catalase (CAT), and glutathione peroxidase (GPx), bolstering the body’s defense against oxidative damage [143]. Ginger prevents the oxidation of low-density lipoprotein (LDL), a key step in plaque formation and progression [144]. Gingerols also exhibit antiplatelet activity, reducing thrombosis risk associated with atherosclerotic plaques [145].

A randomized controlled trial reported that ginger had anti-atherosclerotic and anti-glycemic properties associated with a significantly decreased Lp(a) and fasting blood sugar in patients with atherosclerosis supplemented with ginger for 60 days. Although the levels of cholesterol, TG, LDL, and very-low-density lipoprotein (VLDL) declined in the ginger group, these changes were not statistically significant, while the level of HDL had no significant increase in the ginger group [146].

## 7. Omega-3 Fatty Acids

The antioxidant and anti-inflammatory effects of omega-3 fatty acids (ω-3 PUFAs) have been extensively studied in the context of atherosclerosis. Omega-3 fatty acids, primarily eicosapentaenoic acid (EPA) and docosahexaenoic acid (DHA), are known to exert beneficial cardiovascular effects, largely attributed to their ability to modulate inflammation and oxidative stress [147]. One of the primary mechanisms by which omega-3 fatty acids exert their effects is through the modulation of inflammatory pathways. Studies have demonstrated that omega-3 fatty acids can significantly reduce the production of pro-inflammatory cytokines, such as TNF-α and IL-1β [147].

The anti-inflammatory mechanisms of EPA and DHA also involve changes in cell membrane phospholipid fatty acid composition, disruption of lipid rafts, inhibition of the pro-inflammatory transcription factor nuclear factor κB (reducing the expression of inflammatory genes), and activation of the anti-inflammatory transcription factor peroxisome proliferator-activated receptor γ. Animal studies highlight the beneficial effects of EPA and DHA in various inflammatory models, while human trials show their efficacy in managing rheumatoid arthritis and stabilizing advanced atherosclerotic plaques. Additionally, intravenous administration of n-3 fatty acids may reduce inflammation in critically ill patients [147].

Their anti-inflammatory action is further supported by research indicating that omega-3 fatty acids can decrease matrix metalloproteinase-9 (MMP-9) levels, which are implicated in plaque instability and rupture [147]. In addition to their anti-inflammatory properties, omega-3 fatty acids also exhibit antioxidant effects. They can enhance the antioxidant defense system by increasing the levels of endogenous antioxidants and reducing oxidative stress markers [148]. Feuchtner et al. highlighted that omega-3 fatty acids inhibit the development of vulnerable atherosclerotic plaques by reducing lipid particle incorporation into atheroma, thereby mitigating oxidative damage [149]. The presence of omega-3 fatty acids in cell membranes also influences the production of bioactive lipid mediators, such as resolvins and protectins, which play a crucial role in resolving inflammation and protecting against oxidative stress [150]. Epidemiological studies have consistently shown that higher dietary intake of omega-3 fatty acids is associated with a reduced risk of cardiovascular diseases, including atherosclerosis. For example, Nicholls et al. reported that omega-3 supplementation significantly lowered the incidence of major adverse cardiovascular events in high-risk populations [151]. Similarly, Yu et al. conducted a systematic review and meta-analysis that confirmed the protective effects of omega-3 fatty acids on cardiovascular outcomes, emphasizing their role in reducing myocardial infarction and other cardiovascular events [152].

High doses of omega-3 fatty acids have been shown to lower triglyceride levels and improve overall cardiovascular health, particularly in patients with hypertriglyceridemia. This lipid-modulating effect is critical, as elevated triglycerides are a known risk factor for atherosclerosis and cardiovascular diseases [153].

Omega-3 fatty acids supplementation offered direct vasculoprotective effects, evidenced by a reduction in high-risk “lipid-rich” plaques, independent of coronary risk factors and lifestyle factors [149]. These interactions appear to occur in the early stages of coronary atherosclerosis, leading to less vulnerable plaques, potentially through anti-inflammatory pathways and a stabilizing effect on coronary atheroma [154].

The Randomized, Parallel, Controlled, and Double-Blind Clinical Trial performed by Cartolano et al. concluded that omega-3 was effective in the reduction in cardiovascular risk associated with HDL functionality by size improvement and changes in its lipid, antioxidant, and enzyme composition [155].

A systematic review and meta-analysis of randomized clinical trials pointed out that high-dose omega-3 fatty acids significantly slow the progression of atherosclerosis. The results suggest that anti-atherosclerotic properties of high-dose omega-3 fatty acids are one potential mechanism in reducing CVD outcomes [156].

## 8. Vitamins C and E

The antioxidant and anti-inflammatory effects of vitamins C and E have been extensively studied in relation to atherosclerosis, as both vitamins are known for their roles in protecting cells from oxidative stress and modulating inflammatory responses [157].

Vitamin E, particularly in its form as alpha-tocopherol, is recognized for its antioxidant properties, which help to prevent the oxidation of LDL cholesterol [158]. However, the efficacy of vitamin E in reducing atherosclerosis progression has been debated. For instance, Prasad reported that vitamin E supplementation did not regress hypercholesterolemic atherosclerosis in animal models, suggesting that while it may have antioxidant effects, it does not significantly alter disease progression [159]. In contrast, other studies have indicated that vitamin E can reduce oxidative stress and atherosclerosis in specific contexts, such as in low-density lipoprotein receptor-deficient mice [160]. Vitamin C plays a complementary role to vitamin E by regenerating oxidized vitamin E, thereby enhancing its antioxidant capacity. The interaction between these vitamins is crucial; studies have shown that combined supplementation of vitamins C and E can lead to improved antioxidant effects compared to either vitamin alone [161]. For example, the findings of Agarwal et al. suggest that the intake of vitamin C supplements has an adverse association with early atherosclerosis, while dietary vitamin C from food has a protective association. Their results further suggest that the adverse association of vitamin C supplements may be relatively greater among subjects with elevated cholesterol levels [162]. Furthermore, vitamin C has been shown to prevent the downregulation of endothelial nitric oxide synthase (eNOS) expression in hypercholesterolemic conditions, which is vital for maintaining vascular health [163].

The anti-inflammatory effects of vitamins C and E also contribute significantly to their protective roles against atherosclerosis. Vitamin C has been shown to reduce levels of inflammatory markers and improve endothelial function, which is often compromised in atherosclerosis [164]. In a study by Babaev et al., combined deficiency of vitamins C and E worsened early atherosclerosis in apolipoprotein E-deficient mice, indicating that these vitamins play a protective role against inflammation and plaque formation [165]. Additionally, Nespereira et al. reported that vitamins C and E downregulated vascular endothelial growth factor (VEGF) expression, which is associated with inflammation and angiogenesis in atherosclerosis [166]. Clinical trials have yielded mixed results regarding the effectiveness of vitamin supplementation in preventing atherosclerosis. For instance, Salonen et al. found that long-term supplementation with vitamin C slowed down atherosclerotic progression in hypercholesterolemic persons, suggesting that the timing and context of supplementation may be critical [167]. On the other hand, Nunes et al. reported that a cocktail of vitamins C and E had no effect on the rate of restenosis after angioplasty, indicating that antioxidant supplementation may not always translate to clinical benefits [168]. Despite these mixed findings, the consensus remains that vitamins C and E play important roles in reducing oxidative stress and inflammation, which are pivotal in the pathogenesis of atherosclerosis [169]. Vitamin E supplementation increases the resistance of both LDL and HDL to oxidation and increases cholesteryl ester transfer activity [170]. Moreover, vitamin C was also observed to inhibit lipid oxidation in human HDL and to preserve the antioxidant activity associated with this lipoprotein fraction [171]. The synergistic effects of vitamins C and E in enhancing antioxidant defenses and modulating inflammatory responses underscore their potential as therapeutic agents in cardiovascular health. Future research should focus on elucidating the optimal dosages, timing, and dietary sources of these vitamins to maximize their protective effects against atherosclerosis.

## 9. Coenzyme Q10

Coenzyme Q10 (CoQ10), also known as ubiquinone, is a naturally occurring lipid-soluble quinone in the human body, possessing numerous properties, with its most significant being its antioxidative and anti-inflammatory characteristics [172]. Meat, fish, nuts, and some oils are the richest nutritional sources of CoQ10, while much lower levels can be found in most dairy products, vegetables, fruits, and cereals [173].

CoQ10 is a lipid-soluble antioxidant that has been extensively studied for its potential therapeutic benefits in the context of atherosclerosis [84]. One of the key mechanisms by which CoQ10 exerts its antioxidant and anti-inflammatory effects is through its ability to modulate the activity of NF-κB, a transcription factor that plays a central role in the regulation of inflammatory processes [174]. CoQ10 has been shown to inhibit the NF-κB-activating cascade, thereby reducing the production of pro-inflammatory cytokines, such as TNF-α and IL-6 [175]. Additionally, CoQ10 has been found to enhance the activity of antioxidant enzymes, such as superoxide dismutase (SOD), catalase, and glutathione peroxidase, which are crucial for the neutralization of reactive oxygen species and the maintenance of cellular redox balance [176]. This antioxidant effect of CoQ10 can help mitigate the oxidative stress and endothelial dysfunction associated with atherosclerosis [177]. Furthermore, CoQ10 has been reported to improve mitochondrial function and energy production, which can have beneficial implications for cardiovascular health [178]. Impaired mitochondrial function and reduced adenosine triphosphate (ATP) production are known to contribute to the pathogenesis of atherosclerosis, and the ability of CoQ10 to support mitochondrial activity may help alleviate these underlying mechanisms [179]. Several clinical studies have demonstrated the potential of CoQ10 supplementation in reducing cardiovascular risk factors and improving outcomes in patients with coronary artery disease [180]. For example, a randomized controlled trial showed that CoQ10 supplementation (300 mg/day) for 10 years in elderly individuals led to a significant reduction in cardiovascular mortality compared to the placebo group [181]. The multiple mechanisms of action, including the modulation of NF-κB signaling, enhancement of antioxidant enzyme activities, and support of mitochondrial function, make CoQ10 a promising therapeutic candidate for the prevention and treatment of atherosclerosis-related cardiovascular diseases [178,182].

Several clinical studies demonstrated that CoQ10 supplementation has promising effects on lipid profiles by decreasing TC, TGs, and LDL levels and increasing HDL levels [183,184,185].

## 10. Ginseng (*Panax ginseng*)

Ginseng refers specifically to the fleshy roots and rhizomes of the plant; however, the leaf, berry, and other parts of ginseng are also medicinal sources. It contains several valuable non-saponin components, including essential oils, polyacetylenic alcohols, amino acids, peptides, polysaccharides, antioxidants, and vitamins [186].

The role of ginseng, particularly its active components known as ginsenosides, in mitigating atherosclerosis through antioxidant and anti-inflammatory mechanisms has garnered significant attention in recent research. Studies have demonstrated that ginseng polysaccharides can ameliorate oxidative stress and inflammation in various models, including those induced by ethanol and hyperlipidemia [187]. Specifically, the administration of ginseng extracts has been shown to enhance the expression of antioxidant enzymes, thereby reducing oxidative damage to endothelial cells and improving vascular function [188].

In addition to its antioxidant properties, ginseng also exhibits significant anti-inflammatory effects. Research indicates that ginsenoside Ro, a specific ginsenoside, can attenuate inflammation in macrophages by upregulating heme oxygenase-1, which plays a protective role against inflammatory responses [189]. Furthermore, ginseng berry extracts have been shown to prevent atherogenesis by modulating the expression of phase II detoxifying enzymes, which are involved in the cellular response to oxidative stress and inflammation [190]. The anti-inflammatory action of ginseng is further supported by findings that it can inhibit the production of pro-inflammatory mediators such as nitric oxide (NO) and prostaglandin E2 (PGE2), which are implicated in the inflammatory processes associated with atherosclerosis [191].

The activation of nuclear factor erythroid 2-related factor 2 (Nrf2) by ginseng compounds has been identified as a key mechanism through which ginseng exerts its protective effects against inflammation and oxidative stress in cardiovascular contexts [192].

Moreover, ginseng has been reported to influence lipid metabolism, which is integral to its anti-atherosclerotic effects. Studies have shown that ginseng can lower TC and triglyceride levels in hyperlipidemic models, thereby reducing the lipid burden that contributes to atherosclerosis [191]. The modulation of lipid profiles, combined with its antioxidant and anti-inflammatory properties, positions ginseng as a possible multifaceted agent in the prevention and management of atherosclerosis.

A randomized controlled trial (PEGASUS trial) that evaluated the preventive effects of ginseng against atherosclerosis and subsequent ischemic stroke concluded that ginseng did not demonstrate significant effects compared to placebo; however, none of the patients treated with ginseng experienced ischemic events. Long-term studies involving larger patient populations are needed to further evaluate the effects of ginseng [193].

## 11. Chinese Skullcap (*Scutellaria baicalensis*)

*Scutellaria baicalensis*, commonly known as Baikal skullcap, is a traditional Chinese medicinal herb recognized for its diverse pharmacological properties, particularly its antioxidant and anti-inflammatory effects [194].

The primary bioactive compounds in *Scutellaria baicalensis* include flavonoids, such as baicalin, baicalein, and wogonin, which have been extensively studied for their antioxidant properties. Research indicates that these flavonoids can scavenge ROS and enhance the activity of endogenous antioxidant enzymes, thereby reducing oxidative stress in vascular tissues [195]. For instance, baicalein has been shown to inhibit oxidative stress-induced apoptosis in various cell types, suggesting its potential to protect endothelial cells from damage associated with atherosclerosis [196]. Furthermore, studies have demonstrated that the flavonoids in *Scutellaria baicalensis* can modulate signaling pathways related to oxidative stress, such as the nuclear factor erythroid 2-related factor 2 (Nrf2) pathway, which plays a pivotal role in cellular antioxidant defense mechanisms [197].

In addition to its antioxidant effects, *Scutellaria baicalensis* exhibits significant anti-inflammatory properties. The herb has been shown to inhibit the activation of NF-κB and MAPK, both of which are key regulators of inflammatory responses [198]. By suppressing these pathways, *Scutellaria baicalensis* can reduce the expression of pro-inflammatory cytokines and adhesion molecules that contribute to the pathogenesis of atherosclerosis. For example, wogonin has been reported to inhibit the production of TNF-α and IL-6, which are critical mediators of inflammation in atherosclerotic lesions [199].

The anti-atherosclerotic effects of *Scutellaria baicalensis* are further supported by its ability to suppress Ox-LDL-induced cholesterol accumulation, by reducing Ox-LDL uptake through competitive inhibition of the CD36 binding to the epitope structure of oxLDL [200].

## 12. Specific Effects of Bioactive Compounds on Oxidative Stress and Inflammation in Atherosclerosis

**Figure 2:**
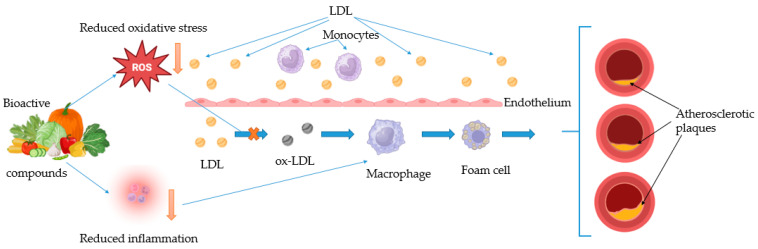
Effects of bioactive compounds on oxidative stress and inflammation in atherosclerosis. Bioactive compounds reduce oxidative stress preventing endothelial dysfunction and LDL oxi-dation. By reducing inflammation they also preserve endothelial function and prevent the recruitment of monocytes and their subsequent transformation into macrophages. The result is a reduction in formation of foam cells and inhibition of the development of atherosclerotic lesions.

Bioactive compounds play a significant role in mitigating oxidative stress and inflammation, key drivers of atherosclerosis. As is presented in Figure 2, the above-mentioned compounds reduce oxidative stress preventing endothelial dysfunction and LDL oxidation. Additionally, they modulate inflammatory pathways by inhibiting pro-inflammatory cytokines, preserving endothelial function, and reducing the recruitment of monocytes and their subsequent transformation into macrophages. The result is a reduction in formation of foam cells and inhibition of the development of atherosclerotic lesions.

Main antioxidant and anti-inflammatory effects of these compounds are summarized in Table 1 and Table 2. Through these mechanisms, bioactive compounds help stabilize atherosclerotic plaques, improve endothelial function, and reduce the progression of atherosclerosis, highlighting their potential as complementary therapies in cardiovascular disease management.

Flavonoids and polyphenols are widely recognized especially for their potent antioxidant properties, which play a crucial role in mitigating oxidative stress and reducing the risk of various diseases, including cardiovascular diseases and cancer.

**Figure 3:**
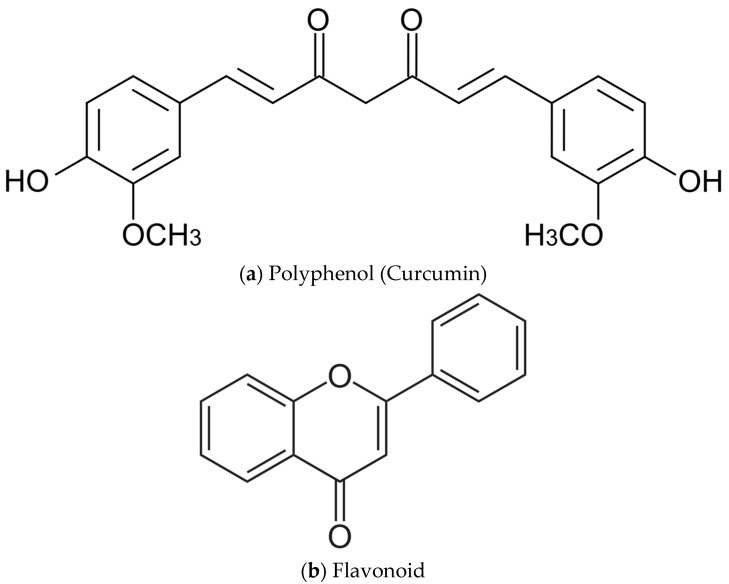
Chemical structure of (**a**) Polyphenol (Curcumin) and (**b**) Flavonoid.

One of the primary mechanisms through which flavonoids and polyphenols exert their antioxidant effects is by scavenging free radicals. This process involves the transfer of hydrogen atoms or electrons to ROS, effectively neutralizing their harmful effects [201]. The antioxidant activity of natural polyphenols is significantly influenced by their structural characteristics, particularly the presence and position of hydroxyl groups (as shown in Figure 3), which enhance their ability to donate electrons and scavenge free radicals [201]. Polyphenols can increase antioxidant capacity by restoring mitochondrial function and regulating antioxidant-responsive signaling pathways, thereby enhancing the overall oxidative stress defense system [202].

Moreover, the interaction of polyphenols with metal ions, such as copper and iron, further contributes to their antioxidant properties [203]. Polyphenols can act as chelators of redox-active transition metals, preventing the formation of harmful free radicals through Fenton-type reactions [204]. This chelation not only reduces oxidative stress but also stabilizes the metal ions, preventing them from catalyzing the production of ROS. The ability of flavonoids to modulate metal ion interactions is crucial in understanding their dual role as antioxidants and potential pro-oxidants under certain conditions [205].

## 13. Possible Limitations of Using Bioactive Compounds as Dietary Supplements and Their Impact on Gut Microbiota and Metabolic Health

### 13.1. Possible Limitations of Using Bioactive Compounds as Dietary Supplements

The bioaccessibility and bioavailability of phytochemicals such as flavonoids, polyphenols, carotenoids, and various herbal compounds are critical factors influencing their health benefits. Bioavailability refers to the proportion of a nutrient or bioactive compound that is absorbed and utilized by the body, while bioaccessibility relates to the fraction that is released from the food matrix during digestion. Both concepts are essential for understanding the efficacy of dietary interventions aimed at disease prevention and health promotion.

The bioavailability of flavonoids and polyphenols is often limited due to poor solubility and rapid metabolism. Studies indicate that the bioavailability of flavonoids can be significantly enhanced through various modifications, such as glycosylation and methylation, which improve their solubility and stability in the gastrointestinal tract [206,207,208]. For instance, glycosylated flavonoids exhibit increased water solubility, which facilitates better absorption [206]. Additionally, structural modifications, such as the creation of flavonoid acetamide derivatives, have been shown to enhance bioavailability by making them more resistant to enzymatic degradation [209].

Food processing techniques also play a crucial role in enhancing the bioaccessibility of flavonoids. By breaking down cell walls and altering the chemical structure of food matrices, processing can increase the release of bioactive compounds during digestion [210]. Moreover, the interaction of flavonoids with dietary fibers can influence their absorption, suggesting that the food matrix is a vital factor in their bioavailability [211].

Similarly to flavonoids, carotenoids face challenges regarding bioavailability due to their lipophilic nature. The presence of dietary fats can enhance the absorption of carotenoids, as they are better solubilized in lipid environments. Furthermore, the matrix in which carotenoids are consumed—whether in whole foods or processed forms—can significantly affect their bioaccessibility and subsequent bioavailability [211].

The bioavailability of active compounds in black cumin (*Nigella sativa*) and ginger (*Zingiber officinale*) can be influenced by their chemical composition and the presence of other dietary components. For example, the bioactive compounds in *Nigella sativa*, such as thymoquinone, exhibit enhanced bioavailability when consumed with fats or oils, which aid in their absorption [212]. Similarly, ginger’s active components, like gingerol, have been shown to have improved bioavailability when consumed in conjunction with other nutrients, a high-fiber diet for example, which facilitates absorption [213].

Omega-3 fatty acids’ bioavailability is influenced by the chemical binding form (free fatty acids bound in ethyl esters, triacylglycerides, or phospholipids), matrix effects (capsule ingestion with concomitant intake of food, fat content in food), or galenic form (i.e., microencapsulation, emulsification) [214]. Research indicated that the bioavailability of omega-3 fatty acids can be enhanced through emulsification processes, which improve their solubility and absorption in the gastrointestinal tract [215].

The bioavailability of Vitamins C and E, as well as Coenzyme Q10, can be affected by their formulation and the presence of other compounds. For instance, the bioavailability of Coenzyme Q10 is notably low when taken orally, but formulations that enhance its solubility, such as nanoemulsions, have been shown to improve absorption significantly [216]. Vitamin C is known to enhance the bioavailability of other nutrients, such as vitamin E and non-heme iron, which may enhance the health effects of vitamin C-containing foods [217].

The bioavailability of ginsenosides (active compounds in ginseng) can be improved through fermentation processes, which enhance their solubility and absorption [218]. For Chinese skullcap, baicalin a bioactive flavonoid has been shown to exhibit enhanced bioavailability when administered in nanocrystals that facilitate its absorption after oral and pulmonary administration [219].

### 13.2. The Impact of Using Bioactive Compounds as Dietary Supplements on Gut Microbiota and Metabolic Health

Flavonoids and polyphenols have been shown to modulate gut microbiota composition, promoting the growth of beneficial bacteria such as *Bifidobacterium* and *Lactobacillus*, which are associated with improved metabolic health, and reducing the number of pathogenic bacteria such as *Escherichia coli*, *Clostridium perfringens*, and *Helicobacter pylori* [220]. The gut microbiota can metabolize polyphenols into bioactive metabolites, enhancing their bioavailability and health benefits [221]. For instance, the interaction between polyphenols and gut microbiota can lead to the production of short-chain fatty acids (SCFAs), which play a crucial role in maintaining gut health, as they act on many cell types to regulate important biological processes, including host metabolism, intestinal function, and immunity [220,222].

Carotenoids have also been implicated in gut health and metabolic regulation. These compounds are known to improve metabolic parameters. Recent studies suggest that carotenoids can influence gut microbiota, as supplementation with β-carotene increased the composition of the abundance of *Bacteroidetes* and *Proteobacteria* and decreased the abundance of harmful bacteria such as *Dialister* and *Enterobacter*, which corroborates the positive effects of this carotenoid in intestinal health [223]. The bioavailability of carotenoids is enhanced when consumed with fats, which can also affect gut microbiota by altering the lipid composition of the gut environment [224].

Herbal remedies using ginger and black cumin have shown potential in modulating gut microbiota and improving metabolic health [225,226]. The bioactive compounds of ginger, such as gingerols and shogaols, were observed to modulate the fecal microbiota structure and promote the growth of some beneficial bacterial populations, such as *Bifidobacterium* and *Enterococcus* [227]. Similarly, *Nigella sativa* has been shown to protect the intestinal mucosa and suppress the growth of potentially harmful gut microbiota [225].

Omega-3 fatty acids can promote the growth of beneficial gut bacteria while reducing the abundance of pro-inflammatory microbes. This modulation of gut microbiota can lead to improved insulin sensitivity and lipid metabolism, thereby supporting overall metabolic health [228].

Vitamins C and E, along with Coenzyme Q10, are vital antioxidants that have been linked to metabolic health through their effects on gut microbiota. Coenzyme Q10, in particular, has been shown to lead to a modification of the taxonomic composition of the gut microbiota, which can enhance its bioavailability and efficacy in metabolic regulation [229]. Furthermore, the interaction between these vitamins and gut microbiota may enhance their bioactive effects, contributing to better metabolic outcomes [229].

Research indicates that also ginseng and Chinese skullcap can modulate gut microbiota, leading to improved metabolic health outcomes [230,231].

## 14. Conclusions

Atherosclerosis is driven by complex interactions between oxidative stress, inflammation, and lipid dysregulation, underscoring the need for multifaceted therapeutic approaches. Natural bioactive compounds exhibit significant antioxidant and anti-inflammatory properties that target key molecular mechanisms involved in atherogenesis. Evidence from preclinical and clinical studies highlights their potential to reduce oxidative stress, suppress pro-inflammatory pathways, stabilize atherosclerotic plaques, and improve vascular function.

Integrating natural bioactive compounds into dietary and therapeutic strategies holds great promise as a complementary approach to traditional atherosclerosis management. Possible limitations of using bioactive compounds as dietary supplements are related to their reduced bioavailability. Continued research and innovation in this area could lead to novel, sustainable solutions for reducing cardiovascular risk and promoting vascular health.

## Figures and Tables

**Table 1:** Main antioxidant effects of different bioactive compounds in atherosclerosis.

Upregulating Antioxidant Enzymes	Neutralization of Free Radicals	Reducing the Production of Reactive Oxygen Species (ROS)	Inhibition of Lipid Peroxidation
Flavonoids Polyphenols Ginger Omega-3 fatty acids Vitamins C and E Coenzyme Q10 Ginseng Chinese skullcap	Carotenoids Coenzyme Q10	Flavonoids Polyphenols Carotenoids Black cumin Chinese skullcap	Polyphenols Back cumin Vitamin C

**Table 2:** Main anti-inflammatory effects of different bioactive compounds in atherosclerosis.

Inhibition of Pro-Inflammatory Cytokines	Modulation of Nuclear Factor-κB (NF-κB)	Modulation of Mitogen-Activated Protein Kinase (MAPK)	Decrease in Inflammation Markers
Flavonoids Polyphenols Carotenoids Black cumin Ginger Omega-3 fatty acids Coenzyme Q10 Chinese skullcap	Flavonoids Polyphenols Carotenoids Black cumin Omega-3 fatty acids Coenzyme Q10 Chinese skullcap	Polyphenols Black cumin Chinese skullcap	Polyphenols Carotenoids Vitamin C Ginseng

## Data Availability

No new data were created or analyzed in this study.
